# Associations of self-management behaviors, depressive symptoms, and glycemic control on cognitive function in rural elderly with type 2 diabetes

**DOI:** 10.3389/fendo.2026.1789318

**Published:** 2026-03-31

**Authors:** Yuting Wang, Yating Qi, Zhixin An, Miaomiao Zhao, Yaqin Zhong, Youjia Wu, Qunhong Wu, Yuexia Gao

**Affiliations:** 1 Department of Health Management, School of Public Health, Nantong University, Nantong, Jiangsu, China; 2 Institute for Health Development, Nantong University, Nantong, Jiangsu, China; 3 Medical School, Nantong University, Nantong, Jiangsu, China

**Keywords:** cognitive function, depressive symptoms, mediation model, self-management behaviors, type 2 diabetes mellitus

## Abstract

**Background:**

The elderly with type 2 diabetes mellitus (T2DM) are often affected by mild cognitive impairment (MCI). However, little is known about the potential mechanisms between the psychological and behavioral factors and MCI among rural Chinese elderly with T2DM. This cohort survey explored the effects of changes in self-management behaviors, depressive symptoms, and glycemic control on MCI.

**Methods:**

This study was conducted from 2019 to 2024 in the rural health clinics in China. At baseline and during follow-up in this cohort study, data on changes in self-management behaviors (the Summary of Diabetes Self-Care Activities, Δ SDSCA), depressive symptoms (the 10-item Center for Epidemiologic Studies Depression Scale, Δ CESD-10), cognitive function (the 30-item Mini-Mental State Examination, MMSE), and glycemic control were assessed. MCI was defined as MMSE scores below education-adjusted cutoffs. Hierarchical multiple regression and mediation model analysis were employed to examine the effects of these variables.

**Results:**

Among 232 participants, 37.07% had MCI at the follow-up stage. Hierarchical multiple regression analysis revealed that improved self-management behaviors (β = 0.233, P < 0.01) significantly predicted better glycemic control. In addition, improved self-management behaviors (increased Δ SDSCA score) (β = 0.220, P < 0.001), less depressive symptoms (decreased Δ CESD-10 score) (β = -0.145, P < 0.05), and better glycemic control (β = 0.143, P < 0.05) were associated with lower risks of MCI. Mediation analysis suggested that better glycemic control partially mediate the effects of improved self-management (increased Δ SDSCA score) on reducing the incidence of MCI (increasing MMSE score) (indirect effect = 0.035, 95% CI [0.006, 0.074]).

**Conclusions:**

Improving self-management behaviors may contribute to more favorable cognitive function by controlling glycemic control. Furthermore, alleviating depressive symptoms may reduce the later incidence of MCI.

## 1 Introduction

Type 2 diabetes mellitus (T2DM) has emerged as a serious health burden in China, accounting for nearly a quarter of the global diabetic population (1, 2). Beyond its metabolic sequelae, T2DM is associated with an elevated risk of vascular complications and microcirculatory dysfunction. These pathophysiological changes are posited to contribute to the development of neurocognitive deficits (3).

Mild cognitive impairment (MCI) represents a cognitive decline greater than expected for an individual’s age and education level, yet not severe enough to interfere significantly with daily activities or meet criteria for dementia (4). T2DM has been identified as a significant independent risk factor for cognitive decline (5). Also, a population-based study in rural China found that diabetes was a significant independent predictor of MCI (6). Among rural older adults with T2DM, the MCI prevalence rate was up to 50.22% (7). Longitudinal evidence suggests that the progression rate from MCI to dementia is 1.5 to 3.0 times higher among individuals with T2DM compared to those without (8). Although hyperglycemia is hypothesized to exacerbate cognitive decline through various mechanisms (9, 10), the long-term cognitive benefits of intensive glycemic control are not conclusively proven. A systematic review found no significant difference in cognitive outcomes between standard and intensive glycemic control over 40–60 months (11), and evidence specific to Chinese populations is limited and inconsistent (12). Therefore, identifying modifiable risk factors and understanding the mechanisms linking T2DM to cognitive impairment are essential for developing strategies to prevent or delay dementia progression.

Effective self-management is foundational to diabetes care and may influence neurological outcomes. Relevant studies have confirmed that lower levels of self-management behaviors have been correlated with poorer cognitive function in patients with T2DM (13, 14). Theoretical models posit that proactive self-management can improve long-term health outcomes (15, 16), yet longitudinal evidence examining how changes in self-management behaviors over time affect MCI incidence remains sparse. Depressive symptoms are highly prevalent in T2DM and aging populations and represent another plausible pathway to cognitive impairment. These symptoms, marked by persistent low mood, anhedonia, and somatic disturbances (17, 18), are a significant public health concern among older adults in China (19). While some longitudinal studies suggest depressive symptoms may be a prodrome or risk factor for cognitive decline (20, 21), others report no significant association (22, 23) or find that risk is confined to specific subgroups (24). Notably, self-management behaviors and depressive symptoms are not independent. Depression is a barrier to effective diabetes self-management, and poor glycemic control resulting from inadequate self-management may exacerbate depressive symptoms (25, 26). Despite this conceptual link, no longitudinal study has investigated how changes in both self-management behaviors and depressive symptoms together influence cognitive outcomes specifically among older adults with T2DM in rural China.

Although self-management behaviors, depressive symptoms, and blood glucose control are all related to the cognitive health of elderly individuals with type 2 diabetes, most evidence is cross-sectional, limiting causal inference regarding how dynamic changes in these factors influence MCI risk. Second, studies focusing on the vulnerable population of rural elderly in China, who may face unique healthcare access and socioeconomic challenges, are scarce. Third, existing longitudinal studies have primarily focused on individual risk factors for cognitive impairment in T2DM, leaving it unclear whether changes in self-management behaviors and depressive symptoms influence cognitive outcomes and whether glycemic control mediates these relationships. This study aims to conduct a longitudinal prospective cohort study among community-dwelling older adults with T2DM in rural China to investigate whether changes in self-management behaviors, depressive symptoms, and glycemic control are associated with the subsequent incidence of MCI. It is expected to provide a reference for the formulation of comprehensive and multifaceted health intervention measures for elderly people with T2DM in rural areas.

## 2 Method

### 2.1 Study design and participants

A prospective longitudinal cohort study was conducted in five health clinics in rural communities in Tongzhou district, Nantong City, southeastern China. Patients diagnosed with T2DM were recruited, and their appointments were scheduled in routine clinics.

At baseline, the inclusion criteria were: (I) have been diagnosed with T2DM for at least one year, based on physician diagnosis; (II) 60 years of age or above; (III) capable of communicating in Mandarin; and (IV) without mild cognitive impairment, defined as an MMSE score above the education-adjusted cutoff.

The included exclusion criteria: (I) diagnosed with severe diabetes complications or functional disorders, including severe cognitive dysfunction (e.g., dementia), blindness, or psychosis; (II) individuals who had emotional disturbances or undergone a traumatic event within the last six months (such as the death of a beloved person, diagnosis of a terminal disease).

Qualified participants were contacted by phone by research assistants to elaborate on the purpose of the current study. Those with interest and eligibility were informed and completed a face-to-face evaluation. All participants provided written informed consent, and the ethics committees of Nantong University approved the study procedures.

**Figure 1:**
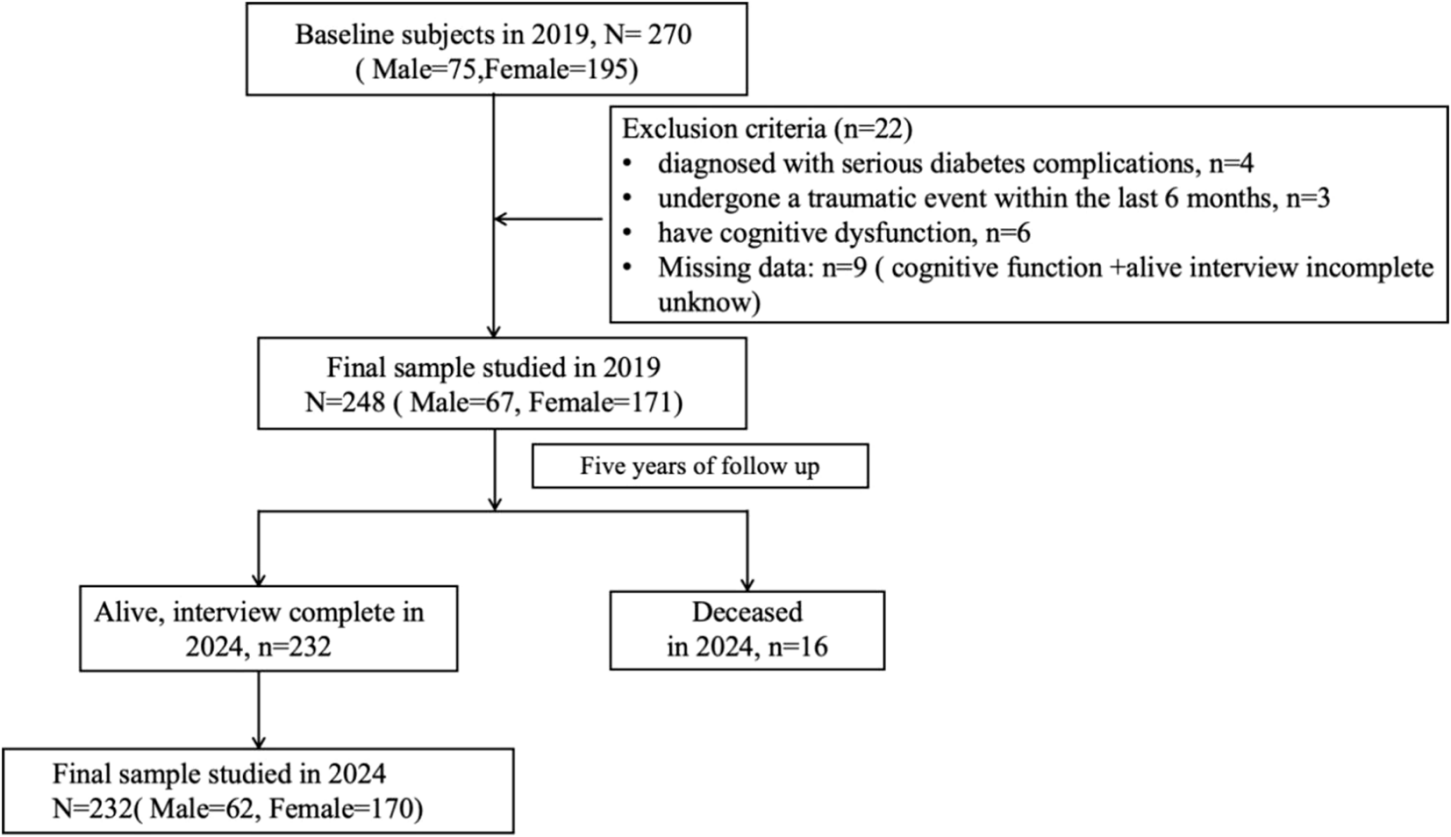
Flow chart of study sample selection.

A cross-sectional survey was conducted at baseline from April to May 2019 (T1). Subsequently, these participants were followed up 5 years after the baseline survey (T2). This study analyzed data from the cohorts surveyed in 2019 and 2024; details of both cohorts are shown in **Figure 1**. Eventually, 232 participants were followed up from April to May 2024 after the baseline survey, with the follow-up rate being 93.55%.

### 2.2 Measures

#### 2.2.1 Changes in self-management behaviors

The diabetes self-management behaviors were evaluated using the Summary of Diabetes Self-Care Activities (SDSCA) (27) The respondents were asked to report the number of days they engaged in routine self-management activities over the past week, which included diet, exercise, blood-glucose testing, foot care, medication taking, and smoking. The SDSCA score was calculated as the sum of the respondents’ answers, with each score rated on a Likert-7 scale, and the higher scores represent a greater level of self-management behaviors. In this sample, Cronbach’s α was 0.80 at T1, and Cronbach’s α was 0.82 at T2, indicating good internal consistency. The variation in self-management behaviors was computed by subtracting the baseline score from the follow-up score for the total SDSCA (Δ SDSCA score), with greater values reflecting more substantial improvements.

#### 2.2.2 Changes in depressive symptoms

Depressive symptoms were assessed using the short form of the 10-item Center for Epidemiologic Studies Depression Scale (CESD0-10) (28), which evaluates the frequency of depressive symptoms experienced by individuals over the previous week, using a 4-point Likert scale that ranges from 1 (0–1 day) to 4 (5–7 days). The total scores on the CESD-10 can range from 1 to 30, and scores of 10 or higher were classified as exhibiting depressive symptoms (29). In the present study, Cronbach’s alpha was found to be 0.88 and 0.90, respectively, indicating strong internal consistency. Changes in depressive symptoms were calculated by variations of the total CESD-10 score at baseline and follow-up (Δ CESD-10 score), with lower differences indicating less severe depressive symptoms.

#### 2.2.3 Glycemic control

Glycemic control was assessed by the fasting plasma glucose (FPG) level, which could be widely accessible in primary care clinics where we conducted the current study. According to the glycemic control recommendation based on the Guideline for the Management of Diabetes Mellitus in the Elderly in China (2024 Edition) (30), FPG<7.2 mmol/L (130 mg/dL) indicated optimal glycemic control, and FPG ≥ 7.2 mmol/L (130 mg/dL) indicated poor glycemic control. FPG data were measured at baseline and follow-up, and changes in glycemic control between baseline and follow-up were classified as 0) maintaining poor (poor to poor), 1) worsening (optimal to poor), 2) improved (poor to optimal), and 3) maintaining optimal (optimal to optimal).

#### 2.2.4 Cognitive function

Cognitive function was evaluated at baseline and follow-up using the Mini-Mental State Examination (MMSE) (31), which consisted of 30 points and included orientation, language, memory, attention, calculation, etc. The authors have obtained proper permission for the use of the MMSE for the research granted by Psychological Assessment Resources, Inc., and the permission was attached as **Supplementary Material**. The line dividing normal (non-MCI) and clinical (MCI) is connected to the level of education: illiteracy <17 points; primary school <20 points; middle school or above <24 points were regarded as having cognitive disorders. In this sample, Cronbach’s α was 0.922 at the follow-up visit.

#### 2.2.5 Control variables

Demographic data included age, gender, marital status, and monthly household income at baseline and follow-up. To determine the association between changes in self-management behaviors, depressive symptoms, and cognitive function, we adjusted for the following potential confounding factors: age (continuous variable), gender (male or female), marital status (never married, married and living together, married but separated, widowed), Monthly household income (less than 2000 yuan, 2000~4000 yuan, 4000~6000 yuan, 6000~8000 yuan, 8000~10, 000 yuan, and more than 10, 000 yuan).

### 2.3 Common method bias

The behavioral and cognitive data were simultaneously collected through a self-rating and reporting questionnaire. Therefore, common method bias (CMB) was a potential issue. In the current study, Harman’s one-factor test was conducted to examine CMB (32), which consisted of implementing statistical control by carrying out a principal component analysis with varimax rotation for all variables. In our study, less than 50 was explained by the first factor, suggesting that CMB does not constitute a problem. Harman’s one-factor test showed that the first factor accounted for 23.28% of the covariance, and three factors had eigenvalues greater than one. As a result, no significant CMB exists in our data.

### 2.4 Data analysis

The data were analyzed using SPSS 27.0, with a significance level set at α = 0.05 for all two-tailed tests. Descriptive statistics, including χ² tests and t-tests, were performed to compare basic characteristics, changes in self-management behaviors (Δ SDSCA score), variations in depressive symptoms (Δ CESD-10 score), and glycemic control between participants with MCI and non-MCI groups (**Table 1**). Partial correlation analyses were carried out to examine associations among MCI, changes in self-management behaviors (Δ SDSCA score), changes in depressive symptoms (Δ CESD-10 score), and glycemic control after controlling for covariates (**Supplementary Table 1**).

**Table 1:** Characteristics of T2DM patients with MCI and non-MCI at follow-up.

Characteristics	Non-MCI (n=146)	MCI (n=86)	*t/x^2^*
Age	70.84 ± 0.63	74.43 ± 0.87	-3.41^***^
Gender			
Males	45 (72.58)	17 (27.42)	3.38
Females	101 (59.41)	69 (40.59)	
Marital status			
Never married	1 (50.00)	1 (50.00)	1.46
Married and living together	121 (64.02)	68 (35.98)	
Married but separated	1 (33.33)	2 (66.67)	
Widowed	23 (60.53)	15 (39.47)	
Monthly household income			
Less than 2000 yuan	65 (54.62)	54 (45.38)	12.44^*^
2000–4000 yuan	26 (66.67)	13 (33.33)	
4000–6000 yuan	20 (62.50)	12 (37.50)	
6000–8000 yuan	9 (69.23)	4 (30.77)	
8000-10, 000 yuan	12 (85.71)	2 (14.29)	
More than 10, 000 yuan	13 (92.86)	1 (7.14)	
Changes in self-management behaviors (Δ SDSCA score)	2.48 ± 1.12	-5.74 ± 1.53	4.39^***^
Changes in depressive symptoms (Δ CESD-10 score)	2.60 ± 0.61	4.13 ± 0.82	-1.51^*^
Glycemic control			
Maintaining poor	45 (56.25)	35 (43.75)	7.88^*^
Worsening	40 (57.97)	29 (42.03)	
Improved	31 (67.39)	15 (32.61)	
Maintaining optimal	30 (81.08)	7 (18.92)	

*p< 0.05. ***p< 0.001.

To investigate causal relationships, hierarchical multiple regression models were employed. First, we assessed the independent effects of changes in self-management behaviors (Δ SDSCA score) (Model 1) and depressive symptoms (Δ CESD-10 score) (Model 2) on glycemic control after adjusting for covariates (**Table 2**). Second, we explored the independent effects of changes in self-management behaviors (Δ SDSCA score) (Model 1) and depressive symptoms (Δ CESD-10 score) (Model 2) and glycemic control (Model 3), respectively, on MCI after adjusting for covariates (**Table 3**). Finally, a combined model (Model 4) was constructed to predict the incidence of having MCI (MMSE score) using the changes in self-management behaviors (Δ SDSCA score), depressive symptoms (Δ CESD-10 score), and glycemic control as predictor variables. Standardized beta coefficients (β) along with 95% confidence intervals (CI) are reported for all models.

**Table 2:** Hierarchical regression models with glycemic control values as dependent variables.

Variables	Model 1	Model 2	Model 3
Changes in self-management behaviors (Δ SDSCA score)	0.233 (0.102, 0.365) ^**^		0.230 (0.099, 0.363) ^**^
Changes in depressive symptoms (Δ CESD-10 score)		-0.063 (-0.197, 0.070)	-0.048 (-0.179, 0.082)
Adj R^2^	0.033	-0.015	0.031
F	2.58^*^	0.29	2.23^*^

Age, gender, marital status, and monthly household income were controlled. *p< 0.05. **p< 0.01.

**Table 3:** Hierarchical regression models with follow-up MCI values as dependent variables.

Variables	Model 1	Model 2	Model 3	Model 4
Changes in self-management behaviors (Δ SDSCA score)	0.263 (0.148, 0.379) ^***^			0.220 (0.104, 0.336) ^***^
Changes in depressive symptoms (Δ CESD-10 score)		-0.168 (-0.286, -0.051)^***^		-0.145 (-0.257, -0.033) ^*^
Glycemic control			0.201 (0.086, 0.315) ^**^	0.143 (0.030, 0.256) ^*^
Adj R^2^	0.254	0.216	0.229	0.291
F	16.72^***^	13.64^***^	14.65^***^	14.46^***^

Age, gender, marital status, and monthly household income were controlled. *p< 0.05. **p< 0.01. ***p< 0.001.

**Figure 2:**
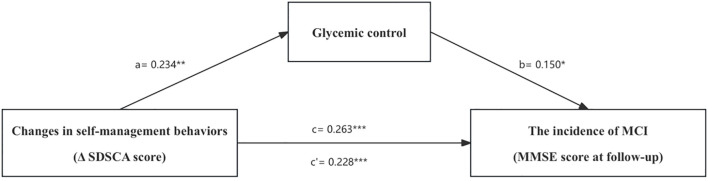
The mediation model. *p< 0.05, **p< 0.01, ***p< 0.001.

Mediation analysis was conducted to test the hypothesized pathways (**Figure 2**) using the PROCESS macro for SPSS, a widely used tool for testing mediation models. Specifically, we evaluated the indirect effects of changes in self-management behaviors (Δ SDSCA score) (Model 1) (X) on the later incidence of MCI (MMSE score at follow-up) (Y) through glycemic control (M) using 95% bias-corrected bootstrap confidence intervals (CIs). As per conventional thresholds, significant mediation was inferred if the 95% CI for the indirect effect excluded zero (33).

## 3 Results

### 3.1 Basic characteristics

Over the 5-year follow-up period, 86 (37.07%) participants suffered from MCI. Chi-squared test and t-test analysis revealed that age and monthly household income were related to the incidence of MCI. In addition, changes in self-management behaviors, depressive symptoms, and glycemic control were associated with the subsequent prevalence of MCI (**Table 1**).

### 3.2 Correlations between changes in self-management behaviors and depressive symptoms, glycemic control, and MCI

Partial correlation coefficients were computed to examine the associations between changes in self-management behaviors, depressive symptoms, glycemic control, and MCI after adjusting for all covariates (**Supplementary Table 1**). The lower incidence of MCI was associated with improved self-management behaviors (higher Δ SDSCA score) (r =0.287, P < 0.001), decreased depressive symptoms (lower Δ CESD-10 score) (r = -0.185, P < 0.01), and better glycemic control (r = 0.225, P < 0.001). Improved self-management behaviors (higher Δ SDSCA score) showed a positive association with glycemic control improvements (r = 0.228, P < 0.001).

### 3.3 The effects of changes in self-management behaviors, depressive symptoms, and glycemic control on MCI by hierarchical regression analysis

As presented in **Table 2**, the hierarchical regression analyses of changes in self-management behaviors and changes in depressive symptoms on glycemic control were carried out and adjusted for all covariates. The results showed that participants with improved self-management behaviors were likely to control glycemic better (β=0.233, 95% CI [0.102, 0.365]). When the changes in self-management behaviors (Δ SDSCA score) and changes in depressive symptoms (Δ CESD-10 score) served as the independent variables together, improved self-management behaviors (Δ increased SDSCA score) were associated with more optimized glycemic control (β=0.230, 95% CI [0.099, 0.363]).

As presented in **Table 3**, the hierarchical regression analyses of changes in self-management behaviors, changes in depressive symptoms, and glycemic control on the later incidence of MCI were conducted and adjusted for all covariates. Participants with improved self-management behaviors (increased Δ SDSCA score) (β=0.263, 95% CI [0.148, 0.379]), less depressive symptoms (decreased Δ CESD-10 score) (β= -0.168, 95% CI [-0.286, -0.051]), and better glycemic control (β= 0.201, 95% CI [0.086, 0.315]) were more likely to have higher MMSE scores, indicating lower incidence of MCI (Models 1-3). The final regression model (Model 4) demonstrated that increasing self-management behaviors (β=0.220), mitigating depressive symptoms (β=-0.145), and optimal glycemic control (β=0.143) were associated with a lower risk of having MCI.

### 3.4 Additional analysis

The results demonstrated a statistically significant association between changes in self-management behaviors (Δ SDSCA score) and glycemic control, which was inversely related to the 5-year incidence of MCI (MMSE score at follow-up). Based on these findings, we hypothesized that glycemic control might mediate the relationship between self-management improvements and the risk of MCI. As shown in **Figure 2**, the mediation analysis confirmed that glycemic control partially explained the link between self-management improvements and the subsequent 5-year incidence of MCI. Improved self-management behaviors (higher Δ SDSCA score) positively affected glycemic control (β =0.234, p<0.01) and the incidence of MCI at follow-up negatively (higher MMSE score) (β =0.228, p<0.001). More optimal glycemic control was associated with a lower incidence of MCI, as indexed by higher MMSE scores (β = 0.150, p<0.01). The indirect effect estimated by the mediation model was notable (β =0.035, 95% CI [0.006, 0.074]), accounting for 13.31% of the total effect (**Table 4**). Specifically, enhanced self-management behaviors could improve glycemic regulation, which in turn exerts protective effects of lowering the likelihood of MCI.

**Table 4:** Analysis of mediation effect.

Pathway	Effect	BootSE	BootLLCI	BootULCI
Total effect	0.263	0.059	0.147	0.379
Direct effect	0.228	0.059	0.111	0.345
Indirect effect	0.035	0.018	0.006	0.074

Age, gender, marital status, and monthly household income were controlled. LLCI, a lower level of the confidence interval; ULCI, an upper level of the confidence interval.

## 4 Discussion

This prospective cohort study provides the empirical evidence examining how longitudinal changes in diabetes self-management behaviors, depressive symptom trajectories, and long-term glycemic regulation collectively influence 5-year MCI incidence in rural Chinese older adults with T2DM. Three key findings emerged from our theoretically guided analyses. First, improvements in self-management behaviors were associated with better glycemic control, supporting behavioral interventions as a pathway to metabolic optimization. Second, sustained self-management, reduced depressive symptoms, and optimal glycemic control against MCI highlight the multifactorial nature of cognitive risk in T2DM and underscore the need for integrated intervention approaches. Third, glycemic control partially mediated the self-management–cognition relationship, suggesting that self-management may protect cognitive function through both direct and indirect pathways.

The main contribution of this study is the identification of a multifactorial protective triad against cognitive decline in rural older adults with T2DM. Sustained improvements in self-management behaviors, reductions in depressive symptoms, and optimal glycemic control collectively mitigated MCI risk through interconnected pathways. Rural older adults face unique challenges compared to their younger or urban counterparts, including limited healthcare access and fewer resources across multiple life domains (34). Interventions that equip rural adults with T2DM with cognitive and behavioral skills for self-management may help protect against cognitive impairment (35). This study found that worsening depressive symptoms predicted higher MCI risk, suggesting that maintaining psychological well-being may preserve cognitive function in this population. One possible explanation is that depressive symptoms contribute to hippocampal volume reduction, increased inflammatory responses, and β-amyloid accumulation, which are linked to cognitive decline (36, 37). Another explanation may be that psychological disorders reduce responsiveness to sensory inputs (38) and their social engagement, thus decreasing brain activeness, reducing cognitive reserve, and impairing cognitive flexibility (39). In addition, metabolically, chronic hyperglycemia and hyperinsulinemia may promote oxidative stress, inflammation, and endothelial damage, accelerating cerebrovascular atherosclerosis and neurodegenerative processes that exacerbate cognitive decline (40).

This study identified a mediating role of glycemic control in the association between changes in self-management behaviors and lower incidence of cognitive impairment. This finding extends the existing literature by demonstrating that behavioral interventions may benefit cognitive health through metabolic pathways in rural older adults with T2DM. On the one hand, relevant studies have indicated that self-management emerges as a cornerstone of diabetes care, with robust evidence supporting its efficiency, cost-effectiveness, and sustainability (41, 42). One possible explanation is that the central skills of self-management, such as problem-solving, decision-making, resource utilization, and action-taking, may enhance patient autonomy and empower individuals to address their health priorities. On the other hand, long-term T2DM often results in a multitude of detrimental outcomes for peripheral systems and neurophysiologic and structural variations in the brain, thereby heightening the risk of MCI (43). In addition, glycemic control mediated 13.31% of the total effect, and the remaining effect may be explained by other potential factors, such as diabetes duration, self-management activities, increased physical activity, and unmeasured psychosocial factors such as health literacy and social support (44, 45). By helping patients maintain glycemic control, effective self-management may mitigate these neurological consequences, decrease cognitive impairment risk, and ultimately improve quality of life in elderly individuals with T2DM. Our findings may offer several practical implications for preventing cognitive decline in older adults with T2DM, particularly in rural settings with limited healthcare resources. First, community health workers in rural clinics could conduct brief glycemic control counseling during routine monthly follow-up visits. These sessions would involve reviewing home glucose monitoring logs, providing feedback on recent readings, and setting simple behavioral goals. This low-cost approach leverages existing personnel and requires minimal additional training. Second, rural health centers could implement a structured diabetes self-management education program delivered by trained nurses. Each session would cover core skills, including blood glucose monitoring, medication adherence, healthy eating within local food contexts, and problem-solving for common barriers. Third, routine depression screening could be integrated into routine diabetes assessments using the two-item Patient Health Questionnaire (PHQ-2). Patients screening positive would receive a brief follow-up assessment by the primary care provider and, if indicated, referral to tele-mental health services or community-based support groups. This screening adds minimal time and cost while enabling early intervention. Several limitations must be acknowledged. First, our study was conducted in rural Nantong, which may limit the generalizability of our findings to young populations or to other areas. Second, we used FPG rather than HbA1c to assess glycemic control. FPG reflects short-term regulation, whereas HbA1c captures chronic hyperglycemia. Consequently, our mediation analysis may underestimate the true role of sustained glycemic control in the self-management–cognition pathway. Third, MMSE has limited sensitivity for detecting MCI, and although we used education-adjusted cutoffs, they were not validated against comprehensive clinical diagnostic assessments in our sample. Fourth, the observational design may limit causal inference. Self-management and glycemic control were measured concurrently at both time points, so our mediation analysis cannot establish temporal precedence. Bidirectional relationships are possible, as poor glycemic control may reduce self-management capacity, and declining cognition may reduce both glycemic control and self-management capacity. Finally, we employed validated instruments (SDSCA scale) to assess self-management behaviors. However, self-reported measures are subject to social desirability bias and recall errors. Moreover, self-reported measures may not capture the full complexity of diabetes self-care, particularly for rural older adults who may receive fragmented or culturally incongruent health advice from multiple sources.

## 5 Conclusion

This 5-year longitudinal cohort study examined how changes in self-management behaviors, depressive symptoms, and glycemic control affect cognitive function in rural Chinese older adults with T2DM. The findings indicate that improved self-management, reduced depressive symptoms, and optimal glycemic control may protect against cognitive impairment, with self-management exerting both direct effects and indirect effects through glycemic control. Our study may provide empirical evidence for developing integrated interventions to preserve cognitive health in this population. Future research should extend these findings by using larger, more diverse samples from multiple rural regions, incorporating objective self-management measures like medication adherence tracking and continuous glucose monitoring, and examining interventions that target behavioral, psychological, and metabolic factors together.

## Data Availability

The raw data supporting the conclusions of this article will be made available by the authors, without undue reservation.
