# Supplementary material for: Associations of self-management behaviors, depressive symptoms, and glycemic control on cognitive function in rural elderly with type 2 diabetes
Source: Front Endocrinol (Lausanne). 2026 Mar 31;17:1789318. doi: 10.3389/fendo.2026.1789318 (PMC13077848; doi:10.3389/fendo.2026.1789318)
Supplement: Supplementary file 1 [file Table1.docx]

Supplementary Table S1 Partial correlation analysis between study variables

| Variables | 1 | 2 | 3 | 4 |
| --- | --- | --- | --- | --- |
| MMSE score at follow-up | 1 |  |  |  |
| Changes in self-management behaviors  (Δ SDSCA score) | 0.287^***^ | 1 |  |  |
| Changes in depressive symptoms  (Δ CESD-10 score) | -0.185^**^ | -0.065 | 1 |  |
| Glycemic control | 0.225^***^ | 0.228^***^ | -0.062 | 1 |

Note: Age, gender, marital status, and monthly household income were controlled. **p*< 0.05.***p*< 0.01.****p*< 0.001.
